# Sacral Neuromodulation Versus Conservative Treatment for Refractory Idiopathic Slow-transit Constipation: The Randomized Clinical No.2-Trial

**DOI:** 10.1097/SLA.0000000000006158

**Published:** 2023-11-23

**Authors:** Stella C.M. Heemskerk, Carmen D. Dirksen, Sander M.J. van Kuijk, Marc A. Benninga, Coen I.M. Baeten, Ad A.M. Masclee, Jarno Melenhorst, Stéphanie O. Breukink

**Affiliations:** * Department of Clinical Epidemiology and Medical Technology Assessment, Maastricht University Medical Center+, Maastricht, the Netherlands; † Care and Public Health Research Institute (CAPHRI), Maastricht University, Maastricht, the Netherlands; ‡ School of Nutrition and Translational Research in Metabolism (NUTRIM), Maastricht University, Maastricht, the Netherlands; § Department of Pediatric Gastroenterology, Emma Children’s Hospital/Amsterdam University Medical Center, Amsterdam, The Netherlands; ∥ Department of Surgery, Groene Hart Hospital, Gouda, The Netherlands; ¶ Division of Hepatology, Maastricht University Medical Center+, Maastricht, The Netherlands; # Department of Surgery, Maastricht University Medical Center+, Maastricht, The Netherlands; ** School for Oncology and Reproduction (GROW), Maastricht University, Maastricht, the Netherlands

**Keywords:** randomized clinical trial, functional constipation, sacral nerve stimulation, constipation management

## Abstract

**Objective:**

Assess the effectiveness of sacral neuromodulation (SNM) versus personalized conservative treatment (PCT) in patients with refractory idiopathic slow-transit constipation (STC).

**Background:**

Evidence on SNM for idiopathic STC is conflicting and of suboptimal methodological quality.

**Methods:**

The No.2-Trial was a multicenter, open-label, pragmatic, randomized trial performed in 2 Dutch hospitals. Sixty-seven patients with idiopathic STC, a defecation frequency <3 per week and refractory (ie, unresponsive) to maximal conservative (nonoperative) treatment were included. Exclusion criteria included outlet obstruction, rectal prolapse, and previous colon surgery. Patients were randomized (3:2) to SNM (n=41) or PCT (n=26) with randomization minimization between February 21, 2017 and March 12, 2020. In SNM patients, an implantable pulse generator was implanted after a successful 4-week test stimulation. PCT patients received conservative treatment such as laxatives or retrograde colonic irrigation. The primary outcome was treatment success (defined as average defecation frequency ≥3 per week) after 6 months. Secondary outcomes included constipation severity, fatigue, quality of life, and adverse events. Analysis was according to intention-to-treat.

**Results:**

After 6 months, 22 (53.7%) patients were successfully treated with SNM versus 1 (3.8%) patient with PCT (odds ratio 36.4, 95% CI 3.4–387.5, *P*=0.003). At 6 months, SNM patients reported lower constipation severity and fatigue scores (*P*<0.001) and improved quality of life compared with PCT (*P*<0.001). Eight serious adverse events (6 SNM, 2 PCT) and 78 adverse events (68 SNM, 10 PCT) were reported.

**Conclusions:**

SNM is a promising surgical treatment option in a homogeneous group of adults and adolescents with refractory idiopathic STC. No.2-Trial registered at ClinicalTrials.gov NCT02961582.

Idiopathic slow-transit constipation (STC) is a subtype of functional constipation (FC), characterized by a delayed colonic transit of feces and the absence of rectal evacuation disorders.^1^ It is a common diagnosis in 15% to 42% of patients with FC but is regularly diagnosed next to a rectal evacuation disorder, like outlet obstruction.^1–3^ It is associated with an impaired physical and mental quality of life, interfered personal daily activities, and work productivity loss.^4–6^

In patients with idiopathic STC refractory to conservative treatment, such as laxatives and retrograde colonic irrigation, surgical interventions may be considered.^7–9^ However, surgical options like subtotal colectomy are invasive, might be ineffective in relieving complaints, and are associated with a risk of (long-term) morbidity and even mortality.^8,10^ Sacral neuromodulation (SNM) is a minimally invasive and safe surgical option delivering electrical stimulation to the sacral nerves, with a low risk of complications in both children and adults.^11^ Although the exact mechanism of action of SNM for functional constipation is poorly understood, it has been shown that suprasensory stimulation induces a colonic motor response by increasing the frequencies of propagating sequences (PS) and high-amplitude propagating sequences (HAPS).^12,13^ Both PS and HAPS are essential for normal colonic transit and defecation but are deficient in idiopathic STC patients.^14^ Hence, the main hypothesized working mechanism of SNM is that it enhances the colonic motor response in patients with idiopathic STC.

Evidence on SNM for FC is conflicting and of suboptimal methodological quality.^15^ As a result, it is not reimbursed by the Dutch health insurance. Three randomized clinical trials (RCT) reported no difference in treatment response between SNM and sham stimulation,^16–18^ whereas observational studies showed success rates up to 52%.^19,20^ These conflicting results might be attributable to heterogeneous patient populations and study designs. Most studies do not differentiate between subtypes of FC, or do not stratify results for STC accordingly.^11,16,18,21^ Only 1 RCT (n=59) exclusively studied refractory idiopathic STC.^17^

The No.2-Trial aimed to provide evidence of good methodological quality on the comparative effectiveness of SNM versus personalized conservative treatment (PCT) in a homogeneous, idiopathic STC population, refractory to conservative treatment. From October 2016 until December 2021, SNM was conditionally reimbursed in the Netherlands for patients participating in the No.2-Trial. The results of this study will be used to reappraise the reimbursement decision by the Dutch Ministry of Health, Welfare, and Sports.^22^ We hypothesized that SNM is an effective treatment modality for patients with idiopathic STC refractory to conservative treatment based on a clinically relevant difference of 30% in treatment success between SNM and PCT.

## METHODS

### Trial Design

The No.2-Trial was a multicenter, pragmatic, open-label, randomized clinical trial. Between February 21, 2017 and March 12, 2020, participants were recruited after referral to 2 Dutch hospitals specialized in SNM for FC: 1 university hospital providing secondary and tertiary care [Maastricht University Medical Center+ (MUMC+), Maastricht], and 1 secondary care hospital [Groene Hart Hospital (GHZ), Gouda]. This study was approved by the Medical Research Ethics Committee (MREC) of the Maastricht University Medical Center+ (MUMC+) and was conducted according to the Declaration of Helsinki. No data safety monitoring board was required by the MREC. For quality assurance purposes, the Clinical Trial Center Maastricht monitored data and safety, and serious adverse events were reported to the MREC and the Dutch Health and Youth Care Inspectorate. All authors had access to the study data and reviewed and approved the final manuscript for publication. This trial is registered at ClinicalTrials.gov (NCT02961582), and the protocol has been published previously.^22^

### Patients

Patients aged 14 to 80 years were deemed eligible when they defecated <3 times per week, fulfilled at least 1 additional item of the Rome-IV criteria for FC,^23^ were diagnosed with idiopathic STC (objectified by radio-opaque marker study, colonic transit time >62 h^24^), and were refractory to conservative treatment. The latter was defined as being unresponsive to maximal conservative (nonoperative) treatment, consisting (of a combination) of lifestyle changes, first-line oral and/or rectal laxatives such as lactulose, macrogol, and bisacodyl, second-line laxatives, such as linaclotide and prucalopride, enemas, and retrograde colonic irrigation. To exclude STC patients with co-existing outlet obstruction, all patients underwent a defecography next to the radio-opaque marker study. Defecating proctograms were assessed by a dedicated abdominal radiologist and colorectal surgeon of the study sites, and patients were excluded in case of anatomic disorders such as (internal) prolapse and/or functional outlet obstruction (eg, not being able to relax pelvic floor muscles). In case of functional outlet obstruction, patients were offered pelvic floor physiotherapy, after which the defecography was repeated, and patients’ eligibility for the study was reassessed. Other exclusion criteria included congenital or organic bowel pathology, rectal prolapse, and previous large bowel or rectal surgery. The complete list of inclusion and exclusion criteria is provided in Supplemental Digital Content Fig. 1, Supplemental Digital Content 1, http://links.lww.com/SLA/E949.

### Sacral Neuromodulation Treatment

Patients were randomly assigned to SNM or PCT, respectively the experimental and control group of this study, in a 3:2 ratio after completion of baseline questionnaires, using the web-based ALEA software (TenALEA consortium, Amsterdam, the Netherlands) operated by research staff. This randomization ratio was chosen to facilitate the inclusion of participants. Randomization by minimization was performed using the study center, age category (14–17 or 18–80 y), and sex as minimization factors. As SNM, a surgical intervention, was compared with conservative treatment, patients and surgeons could not be blinded. Investigators performed the majority of analyses on a data set using random coding for the treatment arm.

Patients allocated to SNM underwent 2 surgical procedures under local or general anesthesia. During the first procedure, a quadripolar tined lead (InterStim 3889, Medtronic Inc., MN, USA) was inserted into the sacral S3 or S4 foramen and connected to an external neurostimulator (InterStim Verify 3051, Medtronic Inc., MN, USA), followed by a 4-week test stimulation period. After a successful test stimulation, defined as an average defecation frequency ≥3 per week based on a 3-week defecation diary, the external neurostimulator was replaced by an implantable pulse generator (IPG) (InterStim 3058, Medtronic Inc., MN, USA). After a nonsuccessful test stimulation, the tined lead was removed, and the patient returned to a personalized treatment algorithm. Detailed operative procedures are published elsewhere.^25^

Patients with an implanted IPG were allowed to use additional conservative (nonoperative) treatment such as (combinations of) oral or rectal laxatives, enemas, or retrograde colonic irrigation during the course of the study period, except for during defecation diary assessments.

### Personalized Conservative Treatment

The PCT group was the control group of this study. Patients allocated to PCT continued care as usual (ie, conservative nonoperative treatment) under the guidance of their referring physician and equal to their treatment before study participation. In general, this consisted of (a combination of) lifestyle changes, first-line oral and/or rectal laxatives such as lactulose, macrogol, and bisacodyl, second-line oral and laxatives such as linaclotide and prucalopride, enemas, and retrograde colonic irrigation. These patients were not offered any additional types of treatment by the physicians of the study team. Treatment of PCT patients was documented in case report forms.

### Outcomes

The primary outcome was treatment success at 6 months follow-up. In the SNM group, due to the period between randomization and the first surgical procedure, the primary outcome was measured 6 months post-lead implantation, whereas, in PCT patients, the primary outcome was measured 6 months post-randomization. Treatment success was defined as an average defecation frequency of ≥3 per week, based on the number of spontaneous bowel movements in the course of a 3-week defecation diary. Spontaneous bowel movements were defined as bowel movements without the use of laxatives, enemas, and/or colonic irrigation. To provide more insight into the effect of SNM on the defecation frequency over time, the difference in the trends of the average defecation frequency per week over time between SNM and PCT was assessed.

Secondary outcomes were the percentage of patients with a 50% reduction in the proportion of defecations with straining and in the proportion of defecations with a sense of incomplete evacuation compared with baseline (3-week defecation diaries); constipation severity (Wexner Constipation Score); fatigue (Dutch fatigue questionnaire); constipation-specific quality of life [Patient Assessment Of Constipation Quality Of Life (PAC-QOL)]; generic health-related (HR) quality of life (QOL) [EuroQol-5D-5L (EQ-5D-5L), ICEpop CAPability measure for Adults (ICECAP-A) in adults (18–80 y), KIDSCREEN-27 in adolescents^14–17^]; and adverse events (reported in case report forms).

### Data Collection

Outcomes were assessed at baseline, 1, 3, and 6 months follow-up. SNM patients with an IPG at 6 months follow-up completed a subsequent outcome measurement at 12 months. An overview of outcome measurements is provided in Supplemental Digital Content Table 1, Supplemental Digital Content 1, http://links.lww.com/SLA/E949. Data were entered into an electronic case report form programmed in the online MACRO electronic data capture system (Clinical Trial Center Maastricht, the Netherlands). Patients were followed from February 21, 2017, until July 31, 2021.

During the diary assessments, patients in both treatment arms were asked to abstain from (additional) conservative (nonoperative) treatments. Patients who did not pass any stool for 3 consecutive days were allowed to use rescue therapy, such as oral and/or rectal laxatives, enemas, and/or colonic irrigation, on every fourth day. This was documented in the diary and was allowed to prevent patients from not passing stools during the 3-week assessment periods. The successful defecation attempts on rescue therapy days were not taken into account when calculating the average defecation frequency per week.

### Statistical Analysis

For the primary outcome, success rates of 35% for SNM and 5% for PCT were estimated. This absolute difference in success rates of 30% was considered clinically relevant and was chosen due to the invasive nature of the treatment and the difference being higher than the estimated general placebo effect of 26% in chronic constipation.^26^ A sample size of 64 patients, with an estimated drop-out rate of 5% (n=3), was required to achieve sufficient statistical power (80%) with a type-I error rate of 5%.

Statistical analyses were performed with SPSS (IBM SPSS Statistics for Windows, Version 26.0. Armonk, NY: IBM Corp) according to intention-to-treat (ITT). For the primary outcome, withdrawn patients were classified as treatment failures, and any violation of the rescue therapy protocol during the diary assessment was managed conservatively: successfully treated patients with SNM who violated the protocol were recoded into treatment failures, whereas successfully treated patients with PCT who violated the protocol remained treatment successors.

Statistical significance was defined as *P* values of 0.05 or less. Multiple imputation with fully conditional specification was used to impute missing data for secondary outcome analyses. The number of imputations was set to 10 and imputed values were drawn using predictive mean matching. Patients without any post-randomization measurements were not taken into account in the analysis of secondary outcomes.

Dichotomous outcomes were expressed as frequencies (%), and continuous outcomes were expressed as means (standard deviation) and medians (interquartile range). Logistic and linear regression, adjusted for randomization minimization factors and the effect of potential unbalanced distributions of baseline variables, were used to analyze differences in secondary outcomes between treatment arms at 6 months for dichotomous and continuous outcomes, respectively. Results from logistic regression analysis were quantified as odds ratio (OR). A multivariable linear mixed-effect model with a random intercept was used for analyzing differences in average defecation frequency per week during the 6 month follow-up period.

Analysis of 12-month outcomes was conducted in the eligible subset of patients with an IPG at 6 months follow-up. Similar to the primary outcome analysis, withdrawn patients were classified as treatment failures. The paired-sample *t*-test was used to compare continuous scores between 6 and 12 months. This study was reported according to the CONSORT statement (see checklist).

## RESULTS

**FIGURE 1:**
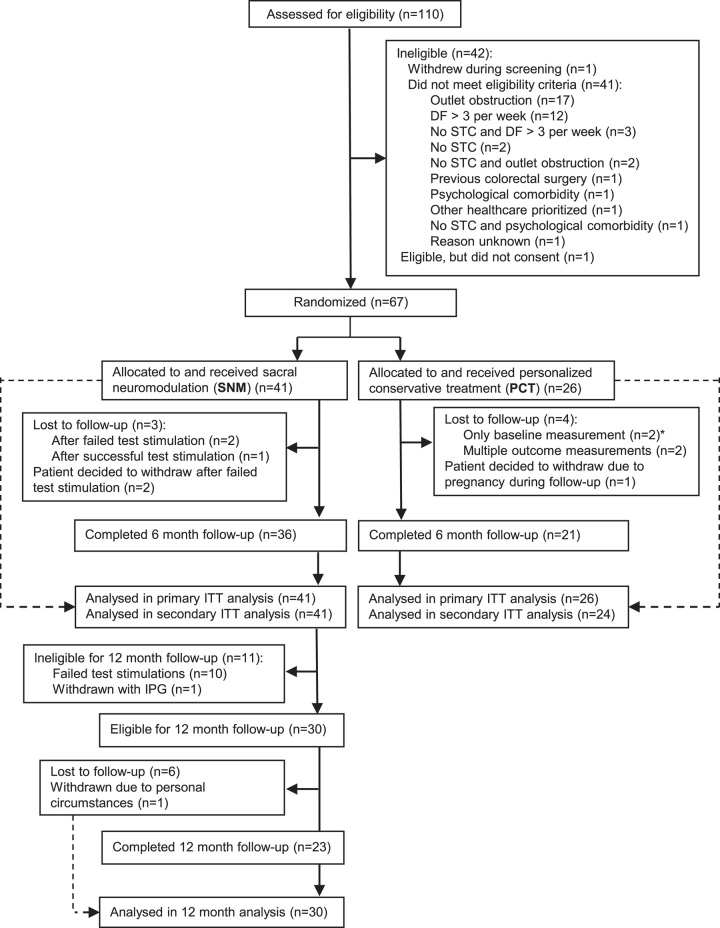
No.2-Trial randomization and follow-up. * Two lost to follow-up patients in the personalized conservative treatment group were excluded from secondary outcome intention-to-treat analyses as no post-randomization measurements were available, and therefore, multiple imputation results were not reliable. DF indicates defecation frequency; IPG, implantable pulse generator; ITT, intention-to-treat; PCT, personalized conservative treatment; SNM, sacral neuromodulation; STC, slow-transit constipation.

Between January 1, 2017, and March 12, 2020, 67 patients (n=35 MUMC+; n=32 GHZ) were enrolled and randomly assigned in a 3:2 ratio to receive SNM (n=41) or PCT (n=26). At the time the 64th patient was included, 6 out of 64 (9.4%) patients were lost to follow-up or had decided to withdraw from the study. The 3 lost to follow-up/withdrawn patients above the estimated drop-out rate of 5% (3 out of 64) were replaced, resulting in 67 patients. Hereafter, patients were no longer replaced. At the end of the study, in total, 10 (14.9%) patients were lost to follow-up (n=3 SNM, n=4 PCT) or had decided to withdraw (n=2 SNM, n=1 PCT) from the study (Fig. 1, CONSORT flow chart). Two of the lost to follow-ups in the PCT group were excluded from secondary outcome analyses due to missing post-randomization measurements. In the primary outcome analysis, these patients were considered treatment failures according to study criteria (ie, withdrawn patients were classified as treatment failures).

### Patient Characteristics

Baseline demographic and clinical characteristics were balanced between groups, except for unbalanced baseline EQ-5D-5L utility scores [mean (SD) SNM 0.514 (0.281) vs. PCT 0.346 (0.266)] (Table 1) (Supplemental Digital Content Table 2, Supplemental Digital Content 1, http://links.lww.com/SLA/E949 for characteristics per study site). Small baseline differences in the same direction as the EQ-5D-5L utility scores were also observed in the PAC-QOL and the adult ICECAP-A scores. In all 41 patients allocated to SNM, tined leads were inserted in S3 (n=38) and S4 (n=3) during the first SNM procedure. In 31 (75.6%) out of these 41 patients, according to study criteria (ie, average defecation frequency ≥ 3 per week), an IPG was implanted after a successful test stimulation (Supplemental Digital Content Fig. 2, Supplemental Digital Content 1, http://links.lww.com/SLA/E949 for SNM patient flow).

**TABLE 1:** Baseline Demographic and Clinical Characteristics of Patients

	SNM (n=41)	PCT (n=26)
Sex		
Female	38 (92.7)	24 (92.3)
Male	3 (7.3)	2 (7.7)
Age (y)	31 (18–52)	32 (19–44)
Adolescents (14–17 y)	10 (24.4)	5 (19.2)
Adults (18–80 y)	31 (75.6)	21 (80.8)
BMI (kg/m^2^)	23.5 (19.9–25.7)	22.9 (21.3–26.3)
Smoking		
No	35 (85.4)	20 (76.9)
Yes	6 (14.6)	6 (23.1)
Previous surgery		
No	19 (46.3)	11 (42.3)
Yes	22 (53.7)	15 (57.7)
Duration of constipation (y)	15 (8-22)	14 (8-25)
Colonic transit time (h)	120 (94-143)	113 (84–132)
Rome-IV criteria (in >25% of defecations…)		
Straining	34 (82.9)	21 (80.8)
Lumpy or hard stools	32 (78.0)	22 (84.6)
Sensation of incomplete evacuation	38 (92.7)	23 (88.5)
Sensation of anorectal obstruction/blockage	23 (56.1)	18 (69.2)
Manual manoeuvres to facilitate	4 (9.8)	3 (11.5)

Values are n (%) and median (IQR).

BMI indicates body mass index; IQR, interquartile range; PCT, personalized conservative treatment; SNM, sacral neuromodulation.

### Primary Outcome

**FIGURE 2:**
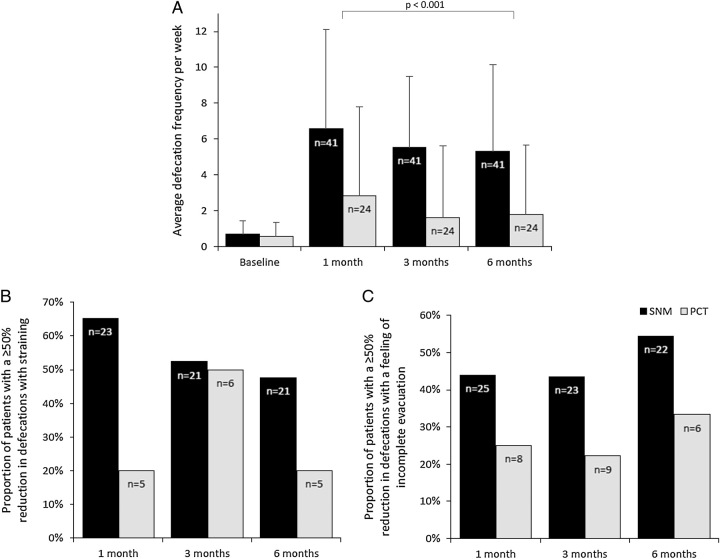
(A–C) Changes in average defecation frequency per week and proportion of defecations associated with straining and a feeling of incomplete evacuation. The “n” in the bars represents the eligible number of patients in the treatment arm for the respective analysis. (A) Mean (SD) defecation frequency after multiple imputations, unadjusted for rescue therapy protocol violations. (B and C) Nonimputed percentages were calculated for the subset of patients with a baseline defecation frequency > 0 and nonmissing follow-up measurements with a defecation frequency > 0. PCT indicates personalized conservative treatment; SNM, sacral neuromodulation.

In the primary ITT analysis, with drop-outs considered as treatment failures, the treatment success rate after 6 months of follow-up differed significantly between the groups: 22 of 41 (53.7%) SNM patients achieved treatment success versus 1 of 26 (3.8%) patients with PCT (OR 36.4, 95% CI 3.4–387.5, *P*=0.003). There was a significant trend over time in the estimated average defecation frequencies per week between both groups in favor of SNM across all outcome measurements (Fig. 2A, regression coefficient 4.54, 95% CI 2.49–6.59, *P*<0.001).

### Secondary Outcomes

Clinically meaningful but not significant between-group differences were observed regarding the proportion of defecations associated with a ≥50% reduction in defecations with straining (Fig. 2B, OR 18.2, 95% CI 0.67–497.50, *P*=0.085) and defecations with a feeling of incomplete evacuation (Fig. 2C, OR 14.9, 95% CI 0.64–346.17, *P*=0.092) after 6 months of follow-up. Significant between-group differences after 6 months of follow-up, in favor of SNM, were observed in Wexner constipation scores, fatigue scores, PAC-QOL scores, EQ-5D-5L utility and VAS scores, and adult ICECAP-A capability scores (Table 2) (Supplemental Digital Content Table 3, Supplemental Digital Content 1, http://links.lww.com/SLA/E949 and 4, Supplemental Digital Content 1, http://links.lww.com/SLA/E949 for results per study site). There were no significant differences between the groups regarding any of the adolescent (HR)QOL KIDSCREEN-27 domain scores. When compared with baseline, all secondary outcomes in SNM patients were significantly improved, whereas in patients with PCT, the majority of the outcomes were not improved (Table 2). An overview of the follow-up data across all outcome measurements (ie, 1, 3, and 6 months follow-up) is provided in Supplemental Digital Content Table 5, Supplemental Digital Content 1, http://links.lww.com/SLA/E949.

**TABLE 2:** Baseline and Six Months Follow-up Secondary Outcome Measures

	Baseline		6 months follow-up			
	SNM (n=41)	PCT (n=26)	SNM (n=41)	PCT (n=24)	Differences in means at 6 mo (SNM vs PCT, 95% CIs)^†^	*P*
Defecation frequency per week	0.71 (0.74)	0.56 (0.78)	5.33 (4.81)^*^	1.64 (3.87)	4.32	< 0.001
	0.33 (0.00–1.33)	0.33 (0.00–0.75)	4.53 (2.32–7.55)	0.33 (0.00–1.00)	(2.01 to 6.64)	—
Wexner Constipation Score^‡^	19.10 (4.39)	19.15 (3.56)	11.81 (4.56)^*^	18.81 (4.36)	−7.29	< 0.001
	19.00 (17.00–22.00)	19.00 (16.75–21.25)	12.00 (8.00–14.90)	18.40 (15.53–21.00)	(−9.72 to −4.87)	—
Fatigue Score^§^	22.33 (4.98)	24.46 (3.28)	16.20 (6.94)^*^	22.60 (5.23)	−6.39	< 0.001
	23.0 (18.00–27.00)	25.50 (21.75–28.00)	16.9 (10.35–21.00)	24.50 (18.78–26.75)	(−9.61 to −3.16)	—
PAC-QOL^∥^	2.63 (0.52)	2.83 (0.56)	1.40 (0.79)^*^	2.48 (0.71)^*^	−1.07	< 0.001
	2.69 (2.34–2.97)	2.74 (2.51–3.20)	1.49 (0.69–1.93)	2.49 (1.80–3.10)	(−1.47 to −0.67)	—
Physical discomfort	2.95 (0.70)	3.07 (0.62)	1.60 (1.00)^*^	2.69 (0.88)	−1.14	< 0.001
	3.00 (2.56–3.50)	3.13 (2.50–3.56)	1.50 (0.75–2.49)	2.70 (2.02–3.44)	(−1.64 to −0.64)	—
Psychosocial discomfort	1.72 (0.79)	2.17 (0.88)	0.82 (0.66)^*^	1.85 (0.84)	−0.96	< 0.001
	1.69 (1.13–2.00)	2.13 (1.63–2.63)	0.75 (0.25–1.16)	1.74 (1.24–2.25)	(−1.34 to −0.57)	—
Worries/concerns	2.36 (0.91)	2.54 (0.91)	1.23 (0.82)^*^	2.21 (0.89)	−0.88	< 0.001
	2.30 (1.55–3.09)	2.69 (1.82–3.29)	1.23 (0.45–1.88)	2.09 (1.45–2.87)	(−1.32 to −0.43)	—
Satisfaction	3.65 (0.37)	3.53 (0.43)	1.97 (1.23)^*^	3.14 (0.92)	−1.30	< 0.001
	3.80 (3.60–3.80)	3.60 (3.20–4.00)	1.98 (0.95–3.00)	3.45 (3.00–3.80)	(−1.90 to −0.71)	—
EQ-5D-5L Utility Score^¶^	0.514 (0.281)	0.346 (0.266)	0.710 (0.234)^*^	0.423 (0.256)	0.243	< 0.001
	0.613 (0.216–0.774)	0.325 (0.150–0.526)	0.762 (0.564–0.861)	0.405 (0.211–0.674)	(0.113 to 0.372)	—
EQ-5D-5L VAS^#^	46.87 (18.29)	41.35 (15.78)	64.31 (18.40)^*^	43.43 (16.26)	19.56	< 0.001
	45.00 (35.00–60.00)	40.00 (30.00–56.25)	65.00 (55.30–80.00)	40.00 (31.25–54.78)	(10.53 to 28.59)	—
ICECAP-A Capability Score^**^	0.73 (0.20)	0.69 (0.19)	0.87 (0.12)^*^	0.71 (0.14)	0.143	< 0.001
	0.80 (0.61–0.92)	0.69 (0.54–0.87)	0.88 (0.82–0.95)	0.70 (0.61–0.84)	(0.064 to 0.221)	—
KIDSCREEN-27^††^						
Physical well-being	30.36 (11.74)	22.46 (10.84)	32.09 (11.35)	27.98 (5.11)	6.75	0.256
	25.07 (22.88–40.45)	20.70 (12.13–33.7)	34.67 (25.96–40.45)	28.13 (23.40–32.22)	(−4.90 to 18.39)	—
Psychosocial well-being	40.01 (8.86)	40.39 (2.06)	43.42 (6.15)	35.94 (5.59)	8.19	0.063
	36.66 (34.88–45.66)	40.39 (38.48–42.31)	41.80 (39.10–49.61)	33.15 (31.94–41.33)	(−0.43 to 16.80)	—
Parent relations and autonomy	51.46 (5.57)	58.30 (5.94)	49.15 (6.33)	54.99 (6.26)	−3.16	0.475
	51.21 (47.23–55.75)	59.06 (52.23–63.99)	49.57 (44.94–53.88)	51.21 (50.34–61.53)	(−11.84 to 5.52)	—
Social support and peers	42.15 (19.82)	50.44 (5.29)	44.14 (13.83)	48.21 (8.49)	−4.02	0.626
	46.92 (22.50–57.83)	49.79 (45.66–55.53)	45.12 (38.90–54.38)	49.79 (40.11–55.53)	(−20.18 to 12.14)	—
School environment	40.44 (11.73)	40.64 (9.05)	47.95 (4.23)	47.05 (8.11)^*^	2.29	0.553
	45.38 (32.58–48.08)	40.72 (31.67–49.58)	48.0 (44.61–48.71)	45.38 (40.81–54.13)	(−5.30 to 9.88)	—

Values are means (SD) and medians (IQR) after multiple imputation.

* Significant (*P*<0.05) change from baseline.

† Linear regression adjusted for randomization factors and unbalanced baseline covariates.

‡ Scores range from 0 (best) to 30 (worst).

§ Scores range from 4 (best) to 28 (worst).

∥ Scores range from 0 (best) to 4 (worst) for both overall and subscale scores.

¶ Scores range from -0.446 (worst) to 1.000 (best) according to the Dutch value set.

# Scores range from 0 (worst) to 100 (best).

** Only measured in adult patients (SNM n=31, PCT n=21 at baseline and n=19 at six months follow-up), scores range from 0.000 (worst) to 1.000 (best) according to the UK value set.

†† Only measured in adolescent patients (SNM n=10, PCT n=5), each domain has its specific scoring range (see KIDSCREEN handbook^23^).

EQ-5D-5L indicates EuroQol-5D-5L; ICECAP-A, ICEpop CAPability measure for Adults; IQR, interquartile range; PAC-QOL, Patient Assessment of Constipation-Quality Of Life; PCT, personalized conservative treatment; SNM, sacral neuromodulation; VAS, Visual Analogue Scale.

**FIGURE 3:**
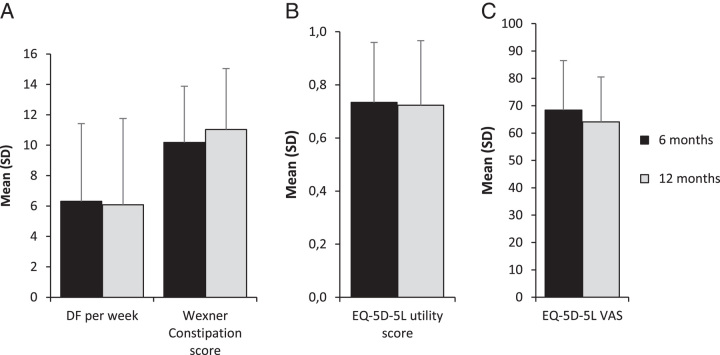
(A–C) Six and 12-month outcome measures for the subset of patients with an implantable pulse generator at 6 months follow-up (n=30). Values are means (SD) after multiple imputations. (A) Mean (SD) defecation frequency per week and Wexner Constipation scores. Wexner Constipation scores range from 0 (best) to 28 (worst). (B) Mean (SD) EQ-5D-5L utility scores ranging from −0.446 (worst) to 1.000 (best) according to the Dutch value set. (C) Mean (SD) EQ-5D-5L VAS scores ranging from 0 (worst) to 100 (best). DF, defecation frequency; EQ-5D-5L, EuroQol-5D-5L; VAS, visual analogue scale.

In the SNM group, 30 patients [mean age 36 y, 27 (90%) females] were eligible for 12 months follow-up, of which 7 were lost to follow-up or withdrawn (Fig. 1). This patient group was equally distributed over the study centers (n=12 MUMC+, n=13 GHZ), and had a similar age category distribution as the ITT SNM group (23% adolescents, 77% adults). After 12 months of follow-up, 14 out of 30 (46.7%) patients achieved treatment success, and the average defecation frequency per week was 6.3 per week (Fig. 3A). A ≥50% reduction in the proportion of defecations with straining and defecations associated with a feeling of incomplete evacuation was reported in, respectively, 73.3 and 62.5% of the patients. No significant differences were observed between 6 and 12-month outcome measurements for the average defecation frequency per week (*P*=0.750), Wexner constipation scores (*P*=0.328), EQ-5D-5L utility scores (*P*=0.839), and EQ-5D-5L VAS scores (*P*=0.218) (Fig. 3A,B,C). When compared with baseline, these secondary outcomes were significantly improved for both 6 and 12-month follow-up.

### Adverse Events

Eight serious adverse events were reported in 7 patients: 6 emergency hospital admissions (n=4 SNM, n=2 PCT) attributed to underlying constipation complaints, 1 hospital admission after a perforated appendix, and COVID-19 infection in the SNM group, not attributed to constipation or SNM, and 1 laryngospasm resulting in desaturation, attributed to the anesthesia during the SNM procedure. Furthermore, 78 adverse events (n=68 SNM, n=10 PCT) were reported in 41 (n=32 SNM, n=8 PCT) patients. The total list of (serious) adverse (device) events is presented in Supplemental Digital Content Table 6, Supplemental Digital Content 1, http://links.lww.com/SLA/E949.

Thirty-two adverse events were typical for SNM. The most reported events included persistent pain/discomfort at the IPG implantation site (n=10), after which 6 IPGs were repositioned; tingling/painful feeling in leg/labia (n=7); and postoperative pain at the wound site (n=3). Three adverse device events were reported: 1 patient underwent 2 lead revisions due to lead defects and 1 patient experienced a software defect of the IPG-programmer. No patients were explanted as a result of adverse events or complete loss of effectiveness. In the PCT group, the most frequently reported adverse events were elective hospital admission for clinical laxation (n=3), colostomy (n=2), and vasovagal complaints (n=2).

## DISCUSSION

This multicenter, open-label, pragmatic RCT in patients with refractory idiopathic STC demonstrated a clinically relevant and significant difference in treatment success after 6 months of follow-up between SNM (53.7%) and PCT (3.8%). When compared with PCT, clinically relevant and statistically significant improvements were observed in constipation severity, fatigue, and disease-specific and generic (HR)QOL scores of the SNM group. Adolescent (HR)QOL scores did not differ significantly between both groups after 6 months of follow-up, which might be attributable to the small number of adolescents included in the study.

Despite the positive effect of SNM, the STC burden was not completely reduced. After 6 months, patients in both groups were considered to be “extremely fatigued” compared with the Dutch population norm data.^27^ Whereas fatigue is recognized as a comorbid symptom in other functional gastrointestinal disorders, it has not yet been reported in studies on idiopathic STC.^28,29^

Three previous crossover, sham-controlled RCTs on SNM for idiopathic (slow-transit) constipation found no difference in efficacy between SNM and sham stimulation, suggesting a placebo response. Firstly, Dinning et al (n=55 patients with STC with permanent SNM) reported no difference in the proportion of patients with a feeling of complete evacuation >2 days/week for at least 2 of 3 weeks, between both suprasensory SNM and sham stimulation (29.6% vs. 20.8%) and subsensory SNM versus sham stimulation (25.4% in both groups).^17^ However, before the crossover phase in this study, 38 (69%) patients were implanted with an IPG despite a failed test stimulation. Secondly, Zerbib et al (n=16 mixed patients with FC with potential co-existing outlet obstruction with permanent SNM) reported no difference in the treatment response rate between subsensory SNM and sham stimulation (60% versus 55%).^16^ However, results were not stratified for STC, and only 16 patients were included in the crossover phase. Thirdly, Yiannakou et al (n=45, mixed patients with FC) reported no difference in the proportion of patients with a ≥0.5 Patient Assessment of Constipation Symptoms score reduction between discriminate and indiscriminate SNM test stimulation responders (60% vs. 57%) at 6 months, with indiscriminate responders defined as responders to sham ± subsensory test stimulation.^18^ This study however ended prematurely (45 of 75 patients) and was designed with a different aim: to assess whether blinded subsensory SNM test stimulation could correctly predict 6-month treatment response after SNM implantation.

Differences between the positive study findings of the No.2-Trial and the findings of previous crossover RCTs may be due to the following reasons. Firstly, in the No.2-Trial we included a highly selected group of patients diagnosed with refractory idiopathic STC without co-existing outlet obstruction. Secondly, all patients who received an IPG implant after the test stimulation phase underwent a thorough selection based on criteria derived from the Rome-IV criteria (ie, average defecation frequency ≥3 per week). Thirdly, we compared SNM with real-world control settings. In our study, this was optimized PCT, reflecting the daily clinical situation for patients with refractory idiopathic STC. The latter might have resulted in the inability to differentiate the overall treatment response from the placebo response. However, it was a deliberate choice to use a non–sham-controlled parallel group design to study the comparative effectiveness of SNM versus PCT, the real-world control setting, in a pragmatic RCT. Ethical considerations (eg, sham-surgery involving complication risk without benefits and the use of expensive devices in a test stimulation setting) played a role when designing the study, as well as subsensory stimulation being regarded suboptimal and therefore an undesirable form of active treatment in a sham-controlled study.^12^ Also, a difference of 49.9% in effectiveness between SNM and PCT in this study might go beyond the estimated general placebo effect of 26% in chronic constipation.^26^

Other differences further complicating the comparison of RCT results include the timing of crossover during the test/permanent SNM phase in the sham-controlled trials and primary and secondary outcome measures used. This heterogeneity impacts the interpretation of results (eg, which patients should be treated with SNM and which outcomes should be assessed during clinical evaluation). Characteristics and results of the RCTs are summarized in Supplemental Digital Content Table 7, Supplemental Digital Content 1, http://links.lww.com/SLA/E949.

In nonrandomized observational studies, SNM success rates range from 31% to 52% in patients with mixed FC subtypes.^19,30,31^ One of the few studies in STC (n=25) reported a significantly increased (spontaneous) DF at 6 months compared with baseline and maintained after 12 months follow-up, and observed improved Wexner and (HR)QOL scores.^19^ Similar to the short-term results, the results on the long-term effectiveness of SNM for FC and idiopathic STC are scarce, contradicting, and of suboptimal quality.^15,19,32^ In our trial, 6-month outcome improvements in patients with SNM were maintained at 12 months, whereas other studies observed a deterioration of effects in the longer term. One long-term follow-up study of a crossover RCT (n=53) reported success rates of 10.8% after one-year follow-up and 5.7% at a median follow-up of 5.7 years.^32^ In contrast, a response rate of 52% was reported for STC at a median follow-up of 60 months.^19^

As stated in the European guideline of FC in adults, (better) patient selection is an unmet need in the evidence landscape of SNM for FC.^8^ The major strength of the No.2-Trial is that the effectiveness of SNM was studied in a homogenous group of patients with refractory idiopathic STC in a setting resembling clinical SNM practice, and measuring a primary outcome that is reported in the majority of STC literature.^28^

This study also had limitations. Firstly, by performing both radio-opaque marker studies and defecographies for all potentially eligible patients, we aimed to include a homogenous group of patients with refractory idiopathic STC without co-existing outlet obstruction. However, despite this being the best way to objectify co-existing outlet obstruction in our daily clinical practice, there might be some overlap. Secondly, asking patients to abstain from medication while completing the defecation diary during all outcome measurements might, for PCT patients in particular, have actively worsened their state. In line with clinical practice, the defecation diary was used as a diagnostic tool to identify eligible patients based on the Rome-IV criteria and, by asking them to abstain from medication, minimize heterogeneity. Hence, to use the identical criteria during the primary outcome measurement in both groups, patients were again asked to abstain from medication. To minimize worsening their state, patients were allowed to use rescue medication. Thirdly, despite randomization, baseline EQ-5D-5L utility scores were unbalanced between the groups. However, this imbalance was adjusted for in the analyses of secondary outcomes. These scores were not influenced by treatment allocation, as baseline questionnaires were administered before randomization. Fourthly, not all patients fully adhered to the medication use protocol, which was, due to trial design, only discovered when the 3-week defecation diaries were completed. For the primary outcome, therefore, violations were handled conservatively by recoding successfully treated SNM patients in violation of the treatment protocol to treatment failure. Fifthly, there might be underreporting of (serious) adverse events in PCT patients due to recall bias after 6 months of follow-up. Lastly, this study had a relatively short follow-up period, with the primary outcome being measured after 6 months of treatment. Even though the majority of SNM patients with an implanted IPG were followed up until 12 months, the long-term effectiveness of SNM in these patients remains unclear.

In conclusion, SNM is a potential, promising treatment option for a select group of patients with refractory idiopathic STC with a follow-up of 6 months in a Dutch real-world clinical setting. Due to the limited evidence of good methodological quality, future studies are required to study the short-term and long-term effectiveness of SNM versus PCT in this patient group, taking into account the current heterogeneity in the available literature.

## Supplementary Material
