# Supplementary material for: Sacral Neuromodulation Versus Conservative Treatment for Refractory Idiopathic Slow-transit Constipation: The Randomized Clinical No.2-Trial
Source: Ann Surg. 2023 Nov 23;279(5):746–54. doi: 10.1097/SLA.0000000000006158 (PMC10997180; doi:10.1097/SLA.0000000000006158)
Supplement: Supplementary file 1 [file sla-279-746-s001.docx]

**Supplemental Digital Content No.2-Trial**

[Figure 1. List of inclusion and exclusion criteria 2](#_Toc143001065)

[Figure 2. No.2-Trial flow chart of patients randomized to the SNM group, undergoing the SNM procedures 3](#_Toc143001066)

[Table 1. Overview of outcome measures and measurement instruments in the No.2-Trial, shown per outcome measurement 4](#_Toc143001067)

[Table 2. Baseline demographic and clinical characteristics of patients stratified by study site 5](#_Toc143001068)

[Table 3. Baseline and six months follow-up secondary outcome measures: Hospital I 6](#_Toc143001069)

[Table 4. Baseline and six months follow-up secondary outcome measures: Hospital II 7](#_Toc143001070)

[Table 5. Overview of follow-up data of secondary outcome measures across all measurement moments in the No.2-Trial 8](#_Toc143001071)

[Table 6. List of (serious) adverse (device) events reported in the No.2-Trial 10](#_Toc143001072)

[Table 7. Overview of randomized controlled trials on sacral neuromodulation for functional constipation 12](#_Toc143001073)

# **Figure 1. List of inclusion and exclusion criteria**

**Inclusion criteria:**

- Aged between 14 and 80 years
- Average defecation frequency of < 3 per week
- Meeting at least one other item of the Rome-IV criteria for functional constipation^1^:
  - In > 25% of the defecations:
    - Straining
    - Lumpy or hard stools
    - Sensation of incomplete evacuation
    - Sensation of anorectal obstruction/blockage
    - Manual maneuvers to facilitate defecation
- Diagnosed with idiopathic slow-transit constipation:
  - Identified by a radio-opaque marker study with a colonic transit time > 62 hours^2^
- Refractory to conservative treatment including (a combination of) oral and rectal laxatives, prokinetics, secretagogues (i.e. linaclotide), and retrograde colonic irrigation

**Exclusion criteria:**

- Diagnosed with outlet obstruction syndrome
  - Objectified with defecography
- Diagnosed with irritable bowel syndrome
- Congenital or organic bowel pathology
- Rectal prolapse
- Anatomical limitations preventing placement of an electrode
- Skin and perineal disease with risk of infection
- Previous large bowel/rectal surgery
- Stoma
- Coexisting neurological disease
- Significant psychological comorbidity
- (Attempted) pregnancy during study follow-up

**References**

1. Lacy BE, Mearin F, Chang L, et al. Bowel Disorders. Gastroenterology 2016;150:1393-1407.
2. Bouchoucha M, Devroede G, Bon C, Raynaud JJ, Bejou B, Benamouzig R. How many segments are necessary to characterize delayed colonic transit time? Int J Colorectal Dis. 2015;30(10):1381-1389.

# **Figure 2. No.2-Trial flow chart of patients randomized to the SNM group, undergoing the SNM procedures**


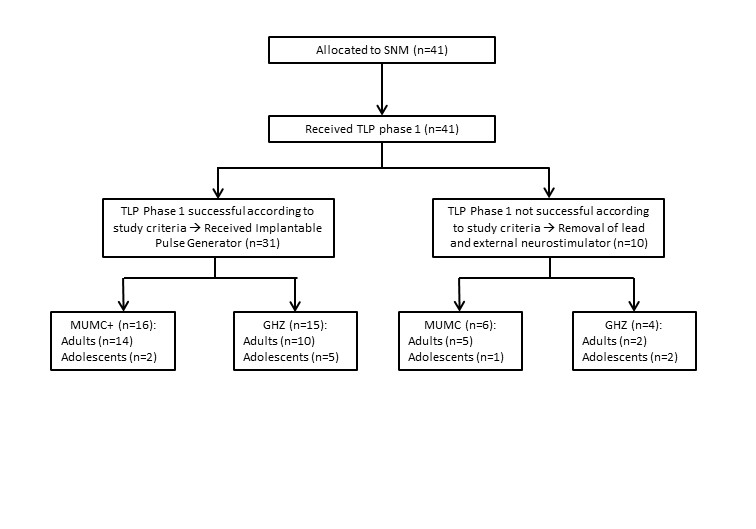


Abbreviations: GHZ: Groene Hart Hospital; MUMC+: Maastricht University Medical Centre+; SNM: sacral neuromodulation.; TLP: tined lead procedure.

# **Table 1. Overview of outcome measures and measurement instruments in the No.2-Trial, shown per outcome measurement**

|  | **Baseline** | **1 month^a^** | **3 months** | **6 months** | **12 months^b^** |
| --- | --- | --- | --- | --- | --- |
| **Treatment success** |  |  |  |  |  |
| 3-week defecation diary | ·· | ·· | ·· | Ѵ | Ѵ |
| **Proportion of patients with 50% reduction in 1) defecations with straining, and 2) defecations with a sense of incomplete evacuation** |  |  |  |  |  |
| 3-week defecation diary | Ѵ | Ѵ | Ѵ | Ѵ | Ѵ |
| **Constipation severity** |  |  |  |  |  |
| Wexner constipation score | Ѵ | Ѵ | Ѵ | Ѵ | Ѵ |
| **Fatigue** |  |  |  |  |  |
| Dutch fatigue questionnaire | Ѵ | Ѵ | Ѵ | Ѵ | ·· |
| **Constipation specific (HR)QOL** |  |  |  |  |  |
| PAC-QOL | Ѵ | Ѵ | Ѵ | Ѵ | ·· |
| **Generic (HR)QOL** |  |  |  |  |  |
| EQ-5D-5L | Ѵ | Ѵ | Ѵ | Ѵ | Ѵ |
| ICECAP-A (in adults only) | Ѵ | Ѵ | Ѵ | Ѵ | ·· |
| KIDSCREEN-27 (in adolescents only) | Ѵ | Ѵ | Ѵ | Ѵ | ·· |
| **(Serious) Adverse events** |  |  |  |  |  |
| Clinician reported | Ѵ | Ѵ | Ѵ | Ѵ | Ѵ |

^a^ Outcome measurements after one month of treatment in the SNM-group were assessed at the end of the four-week test stimulation phase. ^b^ Outcome measurements after 12 months of treatment were only assessed in a subset of patients in the SNM group with an implanted pulse generator at six months follow-up. Abbreviations: EQ-5D-5L: EuroQol-5D-5L; (HR)QOL: (Heath Related) Quality of Life; ICECAP-A: ICEpop CAPability measure for Adults; PAC-QOL: Patient Assessment of Constipation - Quality of Life; SNM: Sacral neuromodulation.

# **Table 2. Baseline demographic and clinical characteristics of patients stratified by study site**

|  | **Hospital I (n=35)** | | | | **Hospital II (n=32)** | | | |
| --- | --- | --- | --- | --- | --- | --- | --- | --- |
|  | **SNM (n=22)** | | **PCT (n=13)** | | **SNM (n=19)** | | **PCT (n=13)** | |
| **Sex** |  |  |  |  |  |  |  |  |
| Female | 21 | (95.5) | 12 | (92.3) | 2 | (10.5) | 1 | (7.7) |
| Male | 1 | (4.5) | 1 | (7.7) | 17 | (89.5) | 12 | (92.3) |
| **Age (years)** |  |  |  |  |  |  |  |  |
| Adolescents (14-17 years) | 3 | (13.6) | 4 | (30.8) | 7 | (36.8) | 1 | (7.7) |
| Adults (18-80 years) | 19 | (86.4) | 9 | (69.2) | 12 | (63.2) | 12 | (92.3) |
| **BMI (kg/m^2^)** | 24.2 | (19.7-26.1) | 23.1 | (21.5-24.3) | 23.4 | (20.9-25.1) | 24.6 | (20.7-26.9) |
| **Smoking** |  |  |  |  |  |  |  |  |
| No | 16 | (72.7) | 10 | (76.9) | 19 | (100) | 10 | (76.9) |
| Yes | 6 | (27.3) | 3 | (23.1) | 0 | (0) | 3 | (23.1) |
| **Previous surgery** |  |  |  |  |  |  |  |  |
| No | 4 | (18.2) | 3 | (23.1) | 15 | (78.9) | 8 | (61.5) |
| Yes | 18 | (81.8) | 10 | (76.9) | 4 | (21.1) | 5 | (38.5) |
| **Duration of constipation (years)** | 20 | (10-24) | 14 | (9-25) | 10 | (5-20) | 10 | (7-25.5) |
| **Colonic transit time (hours)** | 110 | (85-138) | 121 | (88.8-144) | 103 | (96-142) | 115 | (80-127.5) |
| **Rome-IV criteria (in >25% of defecations…)** |  |  |  |  |  |  |  |  |
| Straining | 19 | (86.4) | 19 | (86.4) | 15 | (78.9) | 10 | (76.9) |
| Lumpy or hard stools | 15 | (68.2) | 15 | (68.2) | 17 | (89.5) | 12 | (92.3) |
| Sensation of incomplete evacuation | 22 | (100) | 22 | (100) | 16 | (84.2) | 11 | (84.6) |
| Sensation of anorectal obstruction/blockage | 15 | (68.2) | 15 | (68.2) | 8 | (42.1) | 8 | (61.5) |
| Manual manoeuvres to facilitate | 0 | (0) | 0 | (0) | 4 | (21.1) | 1 | (7.7) |

Values are n (%) and median (IQR). Abbreviations: PCT: personalized conservative treatment; SNM: sacral neuromodulation.

# **Table 3. Baseline and six months follow-up secondary outcome measures: Hospital I**

|  | **Baseline** | | **6 months follow-up** | |
| --- | --- | --- | --- | --- |
|  | **SNM (n=22)** | **PCT (n=13)** | **SNM (n=22)** | **PCT (n=11)*** |
| **Wexner Constipation Score^a^** | 18.23 (3.52) | 18.31 (3.84) | 12.51 (4.13) | 19.22 (4.42) |
|  | 18.50 (16.75-20.50) | 19.00 (15.00-21.50) | 12.45 (10.63-15.05) | 18.40 (16.30-21.00) |
| **Fatigue Score^b^** | 20.82 (5.35) | 24.54 (3.57) | 15.60 (5.67) | 24.55 (3.46) |
|  | 21.00 (16.50-26.25) | 25.00 (22.50-28.00) | 16.65 (10.58-20.00) | 25.00 (23.00-27.00) |
| **PAC-QOL^c^** | 2.73 (0.39) | 2.76 (0.66) | 1.55 (0.73) | 2.64 (0.74) |
|  | 2.73 (2.53-2.96) | 2.68 (2.22-3.30) | 1.66 (0.98-2.11) | 2.49 (2.07-3.22) |
| Physical discomfort | 2.97 (0.66) | 3.00 (0.68) | 1.82 (0.85) | 3.10 (0.68) |
|  | 2.88 (2.50-3.50) | 3.25 (2.25-1.63) | 1.78 (0.99-2.58) | 3.25 (2.50-3.75) |
| Psychosocial discomfort | 1.82 (0.69) | 2.16 (1.06) | 0.95 (0.60) | 1.93 (1.09) |
|  | 1.75 (1.39-2.03) | 2.13 (1.32-3.01) | 0.92 (0.50-1.35) | 1.55 (1.11-2.63) |
| Worries/concerns | 2.52 (0.86) | 2.51 (0.97) | 1.30 (0.78) | 2.22 (0.96) |
|  | 2.50 (1.93-3.18) | 2.64 (1.45-3.46) | 1.65 (0.45-1.87) | 2.00 (1.38-3.00) |
| Satisfaction | 3.70 (0.24) | 3.38 (0.45) | 2.12 (1.16) | 3.31 (0.73) |
|  | 3.80 (3.60-3.80) | 3.40 (3.10-3.80) | 2.30 (1.26-3.10) | 3.60 (3.00-3.80) |
| **EQ-5D-5L Utility Score^d^** | 0.581 (0.240) | 0.344 (0.266) | 0.736 (0.189) | 0.449 (0.210) |
|  | 0.677 (0.408-0.776) | 0.331 (0.099-0.589) | 0.765 (0.604-0.861) | 0.422 (0.349-0.672) |
| **EQ-5D-5L VAS^e^** | 50.14 (16.98) | 41.54 (15.19) | 68.37 (11.61) | 39.84 (9.98) |
|  | 50.00 (35.00-65.00) | 40.00 (30.00-55.00) | 65.13 (59.46-80.00) | 40.00 (35.00-49.66) |
| **ICECAP-A Capability Score^f^** | 0.72 (0.18) | 0.71 (0.19) | 0.85 (0.10) | 0.74 (0.08) |
|  | 0.76 (0.59-0.88) | 0.72 (0.51-0.87) | 0.88 (0.79-0.95) | 0.70 (0.69-0.80) |
| **KIDSCREEN-27^g^** |  |  |  |  |
| Physical well-being | 38.38 (12.41) | 22.90 (12.47) | 35.58 (7.26) | 29.67 (3.65) |
|  | 40.45 (25.07-) | 22.41 (12.13-34.16) | 40.45 (30.57-) | 28.95 (26.61-33.44) |
| Psychosocial well-being | 46.19 (12.68) | 40.72 (2.23) | 44.19 (7.72) | 36.63 (6.20) |
|  | 44.80 (34.26-) | 40.90 (29.50-42.76) | 40.39 (39.10-) | 35.51 (31.33-43.06) |
| Parent relations & autonomy | 56.85 (1.91) | 59.56 (6.04) | 50.75 (7.32) | 56.37 (6.29) |
|  | 55.75 (55.75-) | 61.53 (53.17-64.00) | 47.93 (45.25-) | 55.13 (51.21-62.76) |
| Social support & peers | 49.97 (16.29) | 51.95 (4.70) | 50.33 (9.30) | 49.17 (9.49) |
|  | 49.79 (33.76-) | 51.51 (47.65-56.68) | 53.23 (39.93-) | 51.51 (39.31-56.68) |
| School environment | 48.18 (2.85) | 42.61 (9.14) | 48.08 (0.00) | 49.15 (7.65) |
|  | 48.09 (45.38-) | 44.40 (33.09-50.33) | 48.09 (48.09-48.09) | 46.73 (43.55-57.15) |

Values are means (SD) and medians (IQR) after multiple imputation; * Two patients were excluded from secondary outcome analyses due to missing post-randomization measurements; ^a^ Scores range from 0 (best) to 30 (worst); ^b^ Scores range from 4 (best) to 28 (worst); ^c^ Scores range from 0 (best) to 4 (worst) for both overall and subscale scores; ^d^ Scores range from -0.446 (worst) to 1.000 (best) according to the Dutch value set; ^e^ Scores range from 0 (worst) to 100 (best); ^f^ Only measured in adult patients (SNM n=19, PCT n=9), scores range from 0.000 (worst) to 1.000 (best) according to UK value set; ^g^ Only measured in adolescent patients (SNM n=3, PCT n=4), each domain has its specific scoring range (see KIDSCREEN handbook^23^). Abbreviations: EQ-5D-5L: EuroQol-5D-5L; ICECAP-A: ICEpop CAPability measure for Adults; PAC-QOL: Patient Assessment of Constipation - Quality Of Life; PCT: personalized conservative treatment; SNM: sacral neuromodulation; VAS: visual analogue scale.

# **Table 4. Baseline and six months follow-up secondary outcome measures: Hospital II**

|  | **Baseline** | | **6 months follow-up** | |
| --- | --- | --- | --- | --- |
|  | **SNM (n=19)** | **PCT (n=13)** | **SNM (n=19)** | **PCT (n=13)** |
| **Wexner Constipation Score^a^** | 20.17 (5.17) | 20.00 (3.19) | 11.01 (4.99) | 18.47 (4.42) |
|  | 19.00 (16.75-25.25) | 19.00 (17.50-22.00) | 11.00 (8.00-14.50) | 18.40 (14.65-22.00) |
| **Fatigue Score^b^** | 24.17 (3.87) | 24.38 (3.10) | 16.89 (8.28) | 20.96 (5.97) |
|  | 25.00 (21.25-28.00) | 26.00 (21.00-27.5) | 17.00 (10.00-24.00) | 22.10 (14.90-26.50) |
| **PAC-QOL^c^** | 2.57 (0.64) | 2.89 (0.45) | 1.24 (0.85) | 2.34 (0.67) |
|  | 2.46 (2.09-3.17) | 2.92 (2.63-3.19) | 1.07 (0.66-1.73) | 2.50 (1.64-2.96) |
| Physical discomfort | 2.90 (0.77) | 3.13 (0.58) | 1.35 (1.11) | 2.35 (0.90) |
|  | 3.00 (2.69-3.50) | 3.00 (3.00-3.62) | 1.23 (0.50-2.08) | 2.35 (1.68-3.13) |
| Psychosocial discomfort | 1.61 (0.91) | 2.19 (0.70) | 0.67 (0.71) | 1.78 (0.59) |
|  | 1.57 (0.85-1.96) | 2.13 (1.75-2.63) | 0.50 (0.13-0.99) | 1.81 (1.30-2.13) |
| Worries/concerns | 2.16 (0.96) | 2.56 (0.89) | 1.15 (0.88) | 2.21 (0.86) |
|  | 1.78 (1.43-3.11) | 2.82 (2.05-3.14) | 1.00 (0.45-1.92) | 2.37 (1.48-2.73) |
| Satisfaction | 3.59 (0.49) | 3.68 (0.37) | 1.79 (1.31) | 3.00 (1.06) |
|  | 3.80 (3.40-3.85) | 3.80 (3.30-4.00) | 1.64 (0.80-3.00) | 3.40 (1.89-3.80) |
| **EQ-5D-5L Utility Score^d^** | 0.431 (0.31) | 0.349 (0.276) | 0.679 (0.278) | 0.400 (0.293) |
|  | 0.361 (0.199-0.763) | 0.318 (0.256-0.585) | 0.759 (0.499-0.887) | 0.395 (0.133-0.676) |
| **EQ-5D-5L VAS^e^** | 43.06 (19.49) | 41.15 (16.97) | 59.60 (23.48) | 46.47 (20.04) |
|  | 45.00 (28.75-60.00) | 40.00 (30.00-57.5) | 59.25 (40.00-80.00) | 50.00 (30.00-60.32) |
| **ICECAP-A Capability Score^f^** | 0.75 (0.25) | 0.68 (0.19) | 0.90 (0.14) | 0.70 (0.16) |
|  | 0.82 (0.66-0.92) | 0.67 (0.53-0.87) | 0.95 (0.85-1.00) | 0.70 (0.55-0.86) |
| **KIDSCREEN-27^g^** |  |  |  |  |
| Physical well-being | 26.34 (10.05) | 20.70 | 29.31 (12.07) | 20.70 |
|  | 25.07 (18.56-36.10) | 20.70 | 32.76 (12.13-38.47) |  |
| Psychosocial well-being | 36.93 (5.20) | 39.10 | 43.09 (6.03) | 33.15 |
|  | 36.08 (34.30-39.13) | 39.10 | 43.21 (39.10-48.45) |  |
| Parent relations & autonomy | 48.77 (4.70) | 53.25 | 48.476.36) | 49.47 |
|  | 48.70 (45.33-52.35) | 53.25 | 51.21 (44.02-53.25) |  |
| Social support & peers | 38.25 (21.63) | 44.40 | 41.49 (15.20) | 44.40 |
|  | 45.66 (11.24-57.83) | 44.40 | 43.30 (35.82-53.23) |  |
| School environment | 35.80 (12.84) | 32.79 | 46.72 (5.20) | 38.68 |
|  | 38.68 (23.41-46.73) | 32.79 | 45.92 (43.02-50.58) |  |

Values are means (SD) and medians (IQR) after multiple imputation; ^a^ Scores range from 0 (best) to 30 (worst); ^b^ Scores range from 4 (best) to 28 (worst); ^c^ Scores range from 0 (best) to 4 (worst) for both overall and subscale scores; ^d^ Scores range from -0.446 (worst) to 1.000 (best) according to the Dutch value set; ^e^ Scores range from 0 (worst) to 100 (best); ^f^ Only measured in adult patients (SNM n=12, PCT n=12 at baseline and n=10 at six months follow-up), scores range from 0.000 (worst) to 1.000 (best) according to UK value set; ^g^ Only measured in adolescent patients (SNM n=7, PCT n=1), each domain has its specific scoring range (see KIDSCREEN handbook^23^). Abbreviations: EQ-5D-5L: EuroQol-5D-5L; ICECAP-A: ICEpop CAPability measure for Adults; PAC-QOL: Patient Assessment of Constipation - Quality Of Life; PCT: personalized conservative treatment; SNM: sacral neuromodulation; VAS: visual analogue scale.

# **Table 5. Overview of follow-up data of secondary outcome measures across all measurement moments in the No.2-Trial**

|  | **Baseline** | | **1 month follow-up** | | **3 months follow-up** | | **6 months follow-up** | |
| --- | --- | --- | --- | --- | --- | --- | --- | --- |
|  | **SNM (n=41)** | **PCT (n=26)** | **SNM (n=41)** | **PCT (n=24)** | **SNM (n=41)** | **PCT (n=24)** | **SNM (n=41)** | **PCT (n=24)** |
| **Defecation frequency per week** | 0.71 (0.74) | 0.56 (0.78) | 6.57 (5.54) | 2.66 (4.98) | 5.55 (3.95) | 1.41 (4.02) | 5.33 (4.81) | 1.64 (3.87) |
|  | 0.33 (0.00-1.33) | 0.33 (0.00-0.75) | 5.33 (3.00-8.60) | 0.80 (0.00-3.37) | 5.31 (2.98-7.50) | 0.17 (0.00-0.92) | 4.53 (2.32-7.55) | 0.33 (0.00-1.00) |
| **Wexner Constipation Score^a^** | 19·10 (4·39) | 19·15 (3·56) | 11·71 (5·44) | 18·98 (5·25) | 12·14 (4·05) | 19·68 (3·76) | 11·81 (4·56) | 18·81 (4·36) |
|  | 19·00 (17·00-22·00) | 19·00 (16·75-21·25) | 10·90 (8·00-14·20) | 19·00 (16·33-21·38) | 12·40 (8·45-15·10) | 20·55 (16·13-22·78) | 12·00 (8·00-14·90) | 18·40 (15·53-21·00) |
| **Fatigue Score^b^** | 22·33 (4·98) | 24·46 (3·28) | 17·01 (7·15) | 24·06 (3·70) | 15·92 (6·82) | 23·75 (4·55) | 16·20 (6·94) | 22·60 (5·23) |
|  | 23·0 (18·00-27·00) | 25·50 (21·75-28·00) | 17·22 (9·46-22·50) | 25·00 (21·00-27·75) | 16·70 (10·35-21·00) | 25·00 (21·25-27·75) | 16·9 (10·35-21·00) | 24·50 (18·78-26·75) |
| **PAC-QOL^c^** | 2·63 (0·52) | 2·83 (0·56) | 1·28 (0·81) | 2·69 (0·52) | 1·36 (0·68) | 2·63 (0·72) | 1·40 (0·79) | 2·48 (0·71) |
|  | 2·69 (2·34-2·97) | 2·74 (2·51-3·20) | 1·21 (0·59-1·73) | 2·68 (2·41-2·99) | 1·47 (0·71-1·81) | 2·62 (2·23-3·24) | 1·49 (0·69-1·93) | 2·49 (1·80-3·10) |
| Physical discomfort | 2·95 (0·70) | 3·07 (0·62) | 1·36 (0·94) | 2·89 (0·75) | 1·56 (1·00) | 2·83 (0·89) | 1·60 (1·00) | 2·69 (0·88) |
|  | 3·00 (2·56-3·50) | 3·13 (2·50-3·56) | 1·18 (0·75-1·99) | 3·00 (2·37-3·44) | 1·50 (0·75-2·16) | 2·88 (2·09-3·69) | 1·50 (0·75-2·49) | 2·70 (2·02-3·44) |
| Psychosocial discomfort | 1·72 (0·79) | 2·17 (0·88) | 0·76 (0·65) | 2·12 (0·80) | 0·81 (0·58) | 2·02 (0·88) | 0·82 (0·66) | 1·85 (0·84) |
|  | 1·69 (1·13-2·00) | 2·13 (1·63-2·63) | 0·63 (0·25-1·03) | 2·13 (1·59-2·60) | 0·79 (0·25-1·26) | 1·89 (1·39-2·63) | 0·75 (0·25-1·16) | 1·74 (1·24-2·25) |
| Worries/concerns | 2·36 (0·91) | 2·54 (0·91) | 1·38 (0·82) | 2·40 (0·77) | 1·27 (0·73) | 2·41 (0·81) | 1·23 (0·82) | 2·21 (0·89) |
|  | 2·30 (1·55-3·09) | 2·69 (1·82-3·29) | 1·36 (0·78-1·91) | 2·19 (1·73-2·96) | 1·18 (0·69-1·77) | 2·50 (1·77-3·16) | 1·23 (0·45-1·88) | 2·09 (1·45-2·87) |
| Satisfaction | 3·65 (0·37) | 3·53 (0·43) | 1·63 (1·27) | 3·34 (0·64) | 1·84 (1·15) | 3·38 (0·72) | 1·97 (1·23) | 3·14 (0·92) |
|  | 3·80 (3·60-3·80) | 3·60 (3·20-4·00) | 1·46 (0·50-2·40) | 3·60 (3·00-3·80) | 1·68 (1·08-2·71) | 3·70 (3·20-3·80) | 1·98 (0·95-3·00) | 3·45 (3·00-3·80) |
| **EQ-5D-5L Utility Score^d^** | 0·514 (0·281) | 0·346 (0·266) | 0·676 (0·250) | 0·450 (0·218) | 0·742 (0·203) | 0·435 (0·241) | 0·710 (0·234) | 0·423 (0·256) |
|  | 0·613 (0·216-0·774) | 0·325 (0·150-0·526) | 0·771 (0·614-0·848) | 0·426 (0·283-0·641) | 0·784 (0·639-0·887) | 0·387 (0·235-0·704) | 0·762 (0·564-0·861) | 0·405 (0·211-0·674) |
| **EQ-5D-5L VAS^e^** | 46·87 (18·29) | 41·35 (15·78) | 62·53 (20·33) | 45·56 (16·55) | 66·08 (15·22) | 39·33 (17·92) | 64·31 (18·40) | 43·43 (16·26) |
|  | 45·00 (35·00-60·00) | 40·00 (30·00-56·25) | 65·00 (53·09-80·00) | 49·84 (30·00-60·00) | 70·00 (55·89-80·00) | 37·50 (25·00-52·83) | 65·00 (55·30-80·00) | 40·00 (31·25-54·78) |
| **ICECAP-A Capability Score^f^** | 0·73 (0·20) | 0·69 (0·19) | 0·83 (0·16) | 0·72 (0·16) | 0·86 (0·14) | 0·73 (0·17) | 0·87 (0·12) | 0·71 (0·14) |
|  | 0·80 (0·61-0·92) | 0·69 (0·54-0·87) | 0·88 (0·69-0·95) | 0·70 (0·61-0·85) | 0·91 (0·81-0·97) | 0·76 (0·61-0·87) | 0·88 (0·82-0·95) | 0·70 (0·61-0·84) |
| **KIDSCREEN-27^g^** |  |  |  |  |  |  |  |  |
| Physical well-being | 30·36 (11·74) | 22·46 (10·84) | 33·81 (10·34) | 22·42 (7·96) | 36·21 (15·37) | 25·56 (8·00) | 32·09 (11·35) | 27·98 (5·11) |
|  | 25·07 (22·88-40·45) | 20·70 (12·13-33·7) | 35·60 (27·36-40·56) | 23·49 (14·27-29·50) | 42·53 (18·71-44·73) | 27·13 (18·60-31·24) | 34·67 (25·96-40·45) | 28·13 (23·40-32·22) |
| Psychosocial well-being | 40·01 (8·86) | 40·39 (2·06) | 43·24 (7·87) | 38·9 (4·35) | 45·38 (6·13) | 38·20 (5·77) | 43·42 (6·15) | 35·94 (5·59) |
|  | 36·66 (34·88-45·66) | 40·39 (38·48-42·31) | 44·80 (36·37-50·61) | 37·63 (35·31-43·23) | 44·58 (41·09-53·07) | 37·87 (32·56-44·00) | 41·80 (39·10-49·61) | 33·15 (31·94-41·33) |
| Parent relations & autonomy | 51·46 (5·57) | 58·30 (5·94) | 54·25 (8·62) | 54·12 (3·78) | 53·46 (9·03) | 53·82 (5·36) | 49·15 (6·33) | 54·99 (6·26) |
|  | 51·21 (47·23-55·75) | 59·06 (52·23-63·99) | 52·23 (49·47-59·06) | 53·75 (50·68-57·74) | 50·46 (47·76-55·94) | 53·25 (48·87-59·06) | 49·57 (44·94-53·88) | 51·21 (50·34-61·53) |
|  | **Baseline** | | **1 month follow-up** | | **3 months follow-up** | | **6 months follow-up** | |
|  | **SNM (n=41)** | **PCT (n=26)** | **SNM (n=41)** | **PCT (n=24)** | **SNM (n=41)** | **PCT (n=24)** | **SNM (n=41)** | **PCT (n=24)** |
| Social support & peers | 42·15 (19·82) | 50·44 (5·29) | 43·68 (19·22) | 46·24 (6·29) | 42·16 (17·53) | 48·58 (7·51) | 44·14 (13·83) | 48·21 (8·49) |
|  | 46·92 (22·50-57·83) | 49·79 (45·66-55·53) | 46·93 (31·20-46·42) | 47·22 (40·31-51·69) | 46·43 (32·95-57·83) | 49·79 (41·01-55·53) | 45·12 (38·90-54·38) | 49·79 (40·11-55·53) |
| School environment | 40·44 (11·73) | 40·64 (9·05) | 43·64 (3·66) | 45·55 (7·86) | 45·14 (3·13) | 43·37 (7·91) | 47·95 (4·23) | 47·05 (8·11) |
|  | 45·38 (32·58-48·08) | 40·72 (31·67-49·58) | 43·31 (40·69-46·42) | 45·92 (38·17-52·74) | 45·20 (42·51-48·09) | 48·09 (34·81-49·58) | 48·0 (44·61-48·71) | 45·38 (40·81-54·13) |

Values are adjusted means (SD) and medians (IQR) after multiple imputation; ^a^ scores range from 0 (best) to 30 (worst); ^b^ scores range from 4 (best) to 28 (worst); ^c^ scores range from 0 (best) to 4 (worst) for both overall and subscale scores; ^d^ scores range from -0·446 (worst) to 1·000 (best) according to the Dutch value set; ^e^ scores range from 0 (worst) to 100 (best); ^f^ Only measured in adult patients (SNM n=31, PCT n=21 at baseline and n=19 at six months follow-up), scores range from 0·00 (worst) to 1·00 (best) according to UK value set; ^g^ Only measured in adolescent patients (SNM n=10, PCT n=5), each domain has its specific scoring range (see KIDSCREEN handbook^23^). Abbreviations: EQ-5D-5L: EuroQol-5D-5L; ICECAP-A: ICEpop CAPability measure for Adults; PAC-QOL: Patient Assessment of Constipation - Quality of Life; PCT: personalized conservative treatment; SNM: sacral neuromodulation; VAS: Visual Analogue Scale

# **Table 6. List of (serious) adverse (device) events reported in the No.2-Trial**

|  | **SNM (n=41)** | | **PCT (n=24)** | |
| --- | --- | --- | --- | --- |
|  | **n events** | **n patients*** | **n events** | **n patients*** |
| **Serious adverse events** |  |  |  |  |
| Hospital admission as a result of severe constipation complaints | 4 | 4 | 2 | 1 |
| Hospital admission as a result of perforated appendix + COVID-19 | 1 | 1 | 0 | 0 |
| Laryngospasm, after SNM surgery anaesthesia, resulting in desaturation | 1 | 1 | 0 | 0 |
|  |  |  |  |  |
| **Adverse events typical for SNM** |  |  |  |  |
| Pain and/or discomfort at IPG site requiring surgical IPG repositioning | 6 | 4 | NA | NA |
| Painful and/or tingling feeling in leg and/or labia | 7 | 6 | NA | NA |
| Pain and/or discomfort at IPG site not requiring surgery | 4 | 4 | NA | NA |
| Pain at wound site after surgery | 3 | 3 | NA | NA |
| (Suspected) Wound infection or wound fluid | 3 | 3 | NA | NA |
| Anxiety of infection and/or bleeding after SNM-procedures | 3 | 2 | NA | NA |
| Lead displacement requiring surgical lead revision | 2 | 2 | NA | NA |
| Frequent micturation | 2 | 2 | NA | NA |
| Postoperative nausea/vomiting (SNM-surgery) | 1 | 1 | NA | NA |
| Small electric shocks | 1 | 1 | NA | NA |
|  |  |  |  |  |
| **Adverse device events** |  |  |  |  |
| Lead defect requiring surgical lead revision | 2 | 1 | NA | NA |
| Software defect of the IPG programmer | 1 | 1 | NA | NA |
|  |  |  |  |  |
| **Other adverse events** |  |  |  |  |
| (Stomach) Flu | 5 | 5 | 0 | 0 |
| Hospital admission for clinical laxation due to constipation | 3 | 3 | 3 | 3 |
| Abdominal pain | 3 | 3 | 0 | 0 |
| Vasovagal complaints | 2 | 1 | 2 | 2 |
| Colostomy | 0 | 0 | 2 | 2 |
| Rectal blood loss | 2 | 2 | 0 | 0 |
| Upper tractus digestivus complaints | 1 | 1 | 0 | 0 |
| Extreme constipation complaints | 1 | 1 | 0 | 0 |
| Extreme itch | 1 | 1 | 0 | 0 |
| Feeling ill | 1 | 1 | 0 | 0 |
| Methemoglobin in blood | 1 | 1 | 0 | 0 |
| Muscle tensity in the abdomen | 1 | 1 | 0 | 0 |
| Hip bursitis | 1 | 1 | 0 | 0 |
| Cholecystitis | 1 | 1 | 0 | 0 |
| Bile vomiting | 1 | 1 | 0 | 0 |
| Headache | 1 | 1 | 0 | 0 |
| Pain at stoma site after ileostomy resection prior to SNM-treatment | 1 | 1 | 0 | 0 |
| Throat infection | 1 | 1 | 0 | 0 |
| Torn ankle ligament | 1 | 1 | 0 | 0 |
| Falling without a cause | 1 | 1 | 0 | 0 |
| Persistent fatigue complaints | 1 | 1 | 0 | 0 |
| Uncontrolled flatus | 1 | 1 | 0 | 0 |
| Burn-out | 1 | 1 | 0 | 0 |
| Knee injury | 1 | 1 | 0 | 0 |
| Cataract surgery | 0 | 0 | 1 | 1 |
| Fever | 0 | 0 | 1 | 1 |
| Bladder infection | 0 | 0 | 1 | 1 |

* Some patients experienced multiple (serious) adverse (device) events and therefore, the sum of patients is not equal to the total number of patients who experienced an event mentioned in the manuscript. Abbreviations: NA: not applicable; PCT: personalized conservative treatment; SNM: sacral neuromodulation.

# **Table 7. Overview of randomized controlled trials on sacral neuromodulation for functional constipation**

|  | **Dinning et al., 2015^1^** | **Zerbib et al., 2016^2^** | **Yiannakou et al., 2019^3^** | **Heemskerk et al., 2022**  **(this manuscript)** |
| --- | --- | --- | --- | --- |
| **Study objective** | To evaluate the efficacy of temporary and permanent SNS in the treatment of severe idiopathic slow-transit constipation (STC). | To assess the efficacy of SNS in patients with refractory constipation. | To determine whether blinded  subsensory tined-lead testing could correctly predict  6-month treatment response after SNS implantation | To assess the effectiveness of SNM versus personalized conservative treatment in patients with refractory idiopathic STC. |
| **Study type** | Crossover RCT | Crossover RCT | Crossover RCT | Parallel-group RCT |
| **Study population** | Adults with STC, refractory to conservative treatment. STC was diagnosed by colonic transit scintigraphy: STC if colonic isotope retention of ≥ 20% at 96h.  Patients with radiographic evidence of functional (e.g., pelvic floor dyssynergia) or anatomical (e.g., significant rectocele with retention of contrast, occluding intussusception) impediment were excluded. | Adults with chronic constipation according to the Rome criteria, refractory to conservative treatment.  STC was diagnosed by radio-opaque marker study with an abdominal radiography taken 144h after ingestion of 10 markers on 6 consecutive days: STC if colonic transit time >90h.  Patients with - significant pelvic floor anatomical abnormality (such as rectocele or rectal prolapse) were excluded. | Adults with chronic constipation according to Rome-III criteria, refractory to conservative treatment.  Exclusion of patients with obstructed defecation. | Adolescents and adults (14-80 years) with idiopathic STC refractory to conservative treatment. STC was diagnosed by radio-opaque marker study with an abdominal radiography taken 144h after ingestion of 10 markers on 6 consecutive days: STC if colonic transit time >62h.  Patients with outlet obstruction were excluded. |
| **SNM Intervention** | Permanent implantable pulse generator (IPG) was implanted after a 3-week test stimulation period with peripheral nerve evaluation (PNE). All patients were implanted irrespective of PNE response.  Patients with an implanted IPG entered 2 crossover periods of 8 weeks. Phase 1 consisted of 2 x 3 weeks of subsensory stimulation versus sham-stimulation separated by a 2 week wash-out period. Subsensory stimulation was the perceived stimulation reduced by 0.2V. Phase 2 consisted of 2 x 3 weeks of suprasensory stimulation vs. sham-stimulation separated by a 2 week wash-out period. Suprasensory stimulation was set at 14Hz, 300usec and | Permanent IPG after 3 weeks of PNE in patients who fulfilled at least one of the following criteria: increase in evacuation frequency from less than 2, to ≥3 bowel movements per week; reduction of ≥50 per cent in the number of defecation episodes with straining; or decrease of ≥50 per cent in defecation episodes with a sense of incomplete evacuation.  Two weeks after permanent implantation of IPG, patients entered a 18 week crossover period. This period consisted of 2 x 8 weeks of active versus sham-stimulation separated by 2 week wash-out period. Active stimulation was set at the maximal subsensory threshold. | All patients underwent a 6 week crossover test stimulation period with enhanced PNE/tined lead. The crossover period consisted of 2 x 2 weeks of active versus sham-stimulation separated by a 2 week wash-out period. Active stimulation was set at 75% of the subsensory stimulation threshold.  Permanent IPG was implanted after the 6 week test stimulation/crossover period in all responders with a ≥ 25% improvement TiLTS-VAS score (0-100 visual analogue scale of patients’ self-assessment of the effectiveness of the testing period in improving their symptoms), assessed at the end of each 2-week period including wash-out. | Permanent IPG was implanted after 4 weeks of tined lead test stimulation in the patients with an average defecation frequency ≥3 per week. |
| **Control treatment** | Sham stimulation (pulse width and frequency set to 0). | Sham stimulation (not specified). | Sham stimulation (not specified). | Personalized Conservative Treatment (PCT): patients continued their personalized treatment program |
| **Primary outcome** | Proportion of patients who on more than 2 days per week report a bowel movement associated with a feeling of complete evacuation for at least 2 or 3 weeks during permanent suprasensory stimulation, based on stool diaries. | Proportion of patients with a response during each crossover treatment period (active vs. sham), defined by the same criteria as test stimulation success (see “SNM intervention”). Response was measured during the last 3 weeks of each of the 8-week treatment periods. | Proportion of patients with a ≥ 0,5 reduction in PAC-SYM at 6 months. | Treatment success at 6 months after lead implantation (SNM group) and randomization (control group), defined as an average defecation frequency of ≥ 3 per week |
| **Secondary outcomes** | Treatment response according to primary outcome criteria, but during subsensory stimulation.  Tertiary outcomes:   - PNE as predictor of treatment efficacy following permanent electrode placement; - Effects of SNM on abdominal pain and bloating score, laxative-free days, stool frequency, stool form, and quality of life (SF36) during both supra- and subsensory stimulation. | - Short- and long-term clinical and physiological factors associated with response to temporary and permanent SNS; - Percentage of patients with a response at 1 year; - Effects of SNS on patients’ daily bowel diary items; - Wexner Score; - Effect on quality of life (GIQLI); - Visual analogue score rating bowel habit; - Anorectal manometry parameters; - Colonic transit time. | - Number of the discriminate and indiscriminate responder; - Scores from daily diaries; - Quality of life (PAC-QOL, EQ-VAS, TilTS-VAS, EQ-5D); - Constipation symptoms scores (Cleveland, Wexner). | 6 months after treatment:   - Percentage of patients with a 50% reduction in the proportion of defecations with straining and in the proportion of defecations with a sense of incomplete evacuation compared with baseline (three-week defecation diaries); - Constipation severity (Wexner); - Fatigue (Dutch fatigue questionnaire); - Constipation-specific quality of life (PAC-QOL); - Generic (health-related) quality of life (EQ-5D-5L, ICECAP-A in adults, KIDSCREEN-27 in adolescents).   After 12 months Wexner and EQ5D5L were measured in patients with an implanted IPG. |
| **Total N** | 59 | 36 | 45 | 67 (n=41 SNM, n=26 PCT) |
| **N (SNM PNE/TLP test phase)** | 59: 2 patients were removed from PNE analysis; 16 (28%) out of 57 patients were deemed responders to PNE. | 36 (78% STC) | 45 (67% STC) | 41 |
| **N permanent SNM** | 55 (Phase 1: subsensory vs sham)  53 (Phase 2: suprasensory vs sham) | 20 (% STC not reported) | 27 (% STC not reported) | 31 |
| **Loss to follow-up** | 4 following PNE  2 with permanent IPG | 4 with permanent IPG, after crossover period |  | 5 with SNM  5 with PCT |
| **Results:**  **Primary outcome** | Primary outcome reached by 16 (29.6%) of 54 patients during suprasensory stimulation versus 11 (20.8%) of 53 patients during sham-stimulation (p=0.23); 6 patients responded to both.  When compared with baseline, both suprasensory and sham stimulation caused significant changes in a number of measured characteristics (e.g. feeling of complete evacuation, normal stool, pain and bloating score). | Primary outcome was reached by 12 (60%) of 20 patients during active stimulation versus 11 (55%) of 20 patients during sham-stimulation (p=0.746). | Primary outcome was reached by 15 (57.7%) of 27 patients with an IPG versus 5 (71.4%) patients without an IPG (no statistical testing). | Primary outcome reached by 22 (53.7%) of 41 patients in the SNM group versus 1 (3.8%) of 26 patients in the PCT group (p=0.003). |
| **Results:**  **Secondary outcomes** | Treatment response reached by 14 (25.4%) of 55 patients during both subsensory and sham-stimulation periods (p=0.95).  Tertiary outcomes:  When compared with sham-stimulation, active stimulation had no significant impact on any of the tertiary outcome measures.  When compared with baseline, subsensory and sham-stimulations caused a significant improvement in several characteristics (e.g. feeling of complete evacuation, normal stool, pain and bloating score). | During the crossover period, no differences between active and sham-stimulation periods for stool diary items, Wexner scores, VAS score, or QoL scores.  At 1 year, 11 (55%) of 20 patients with IPG continued to respond.  Compared with baseline, at 1 year SNS was associated with significant improvement in defecation with sensation of incomplete evacuation, number of days with modest to severe pain, Wexner and VAS scores, and quality of life subscores symptoms, physical condition and emotions. | 29 (64.4%) of 45 patients were responders to the testing phase:   - 7 (18% total) were discriminate responders; - 22 (56% total) were indiscriminate responders - There was no significant difference between discriminate and indiscriminate responders in meeting the primary endpoint (p=0.76).   PAC-QOL improved at 12 weeks but deteriorated at 6 months; whereas other scores (Cleveland and Wexner) showed improvement at 6 months. Furthermore, there were small improvements in constipation symptoms among patients with IPG. | After 6 months, no differences between the groups was observed in the proportion of defecations associated with a reduction in defecations with straining and incomplete evacuation.  Significant between-group differences, in favour of SNM were found for Wexner, fatigue, PAC-QOL, EQ-5D-5L utility and VAS, and adult ICECAP-A scores. No differences were found in KIDSCREEN-27 scores in adolescents.  After 12 months, 14 (46.7%) out of 30 eligible patients achieved treatment success. No significant differences were found between 6 and 12 months Wexner and EQ-5D-5L utility and VAS scores. |
| **Adverse events (AE) in SNM patients** | 73 AEs reported:   - 32 (44%) pain at IPG site - 12 (16%) wound infection at implantation site - 4 (5%) leg pain/discomfort - 3 (4%) abdominal pain/discomfort - 17 (23%) urological events - 1 (1%) tachycardia - All n=2 (3%) headache; altered mood | 9 serious AEs reported in 8 patients:   - 2 wound infections - 2 abdominal pain and asthenia - All n=1: electrode wire displacement; sciatica; abdominal pain and asthenia; abdominal pain and anaemia; sinusitis; vagal response   25 AEs (possibly) related to device:   - 5 sciatica/pain at IPG site - 3 wound infection/abcess/haematoma - 4 abdominal pain - All n=2: dysuria, faecal impaction and asthenia - All n=1: fever, anal pain, vaginal infection, anaemia, nausea, faecal incontinence, dehydration | 56 AEs related to trial in 40 (89%) patients:   - 10 infections; 9 led to urgent removal of tined lead during test phase (6/45 (13%)) or IPG (3/27 (11%)). | 6 serious AEs reported in 4 patients:   - 4 hospital admissions: severe constipation complaints - 1 hospital admission: perforated appendix + COVID-19 - 1 laryngospasm after SNM surgery anaesthesia, resulting in desaturation   68 AEs typical for SNM:   - 6 pain at IPG site requiring IPG repositioning - 7 painful leg/labia - 4 pain/discomfort at IPG site - 3 pain at wound site - 3 (suspected) wound infection/wound fluid - 3 anxiety of infection - 2 lead displacement requiring surgical lead revision - 2 frequent micturation - 1 postoperative nausea - 1 small electric shocks - 2 lead defects requiring surgical lead revision - 1 software defect IPG programmer |

Abbreviations: IPG: implantable pulse generator; PCT: personalized conservative treatment; PNE: peripheral nerve evaluation; RCT; randomized controlled trial; SNM: sacral neuromodulation; STC: slow-transit constipation; SNS: sacral nerve stimulation

**References eTable 7:**

1. Dinning PG, Hunt L, Patton V, et al. Treatment efficacy of sacral nerve stimulation in slow transit constipation: a two-phase, double-blind randomized controlled crossover study. Am J Gastroenterol 2015;110:733-40.
2. Zerbib F, Siproudhis L, Lehur PA, et al. Randomized clinical trial of sacral nerve stimulation for refractory constipation. Br J Surg 2017;104:205-213.
3. Yiannakou Y, Etherson K, Close H, et al. A randomized double-blinded sham-controlled cross-over trial of tined-lead sacral nerve stimulation testing for chronic constipation. Eur J Gastroenterol Hepatol 2019;31:653-660
